# Supplementary material for: Engineered endosymbionts that alter mammalian cell surface marker, cytokine and chemokine expression
Source: Commun Biol. 2022 Aug 30;5:888. doi: 10.1038/s42003-022-03851-6 (PMC9427783; doi:10.1038/s42003-022-03851-6)
Supplement: Supplementary file 2 — Supplementary Information [file 42003_2022_3851_MOESM2_ESM.docx]

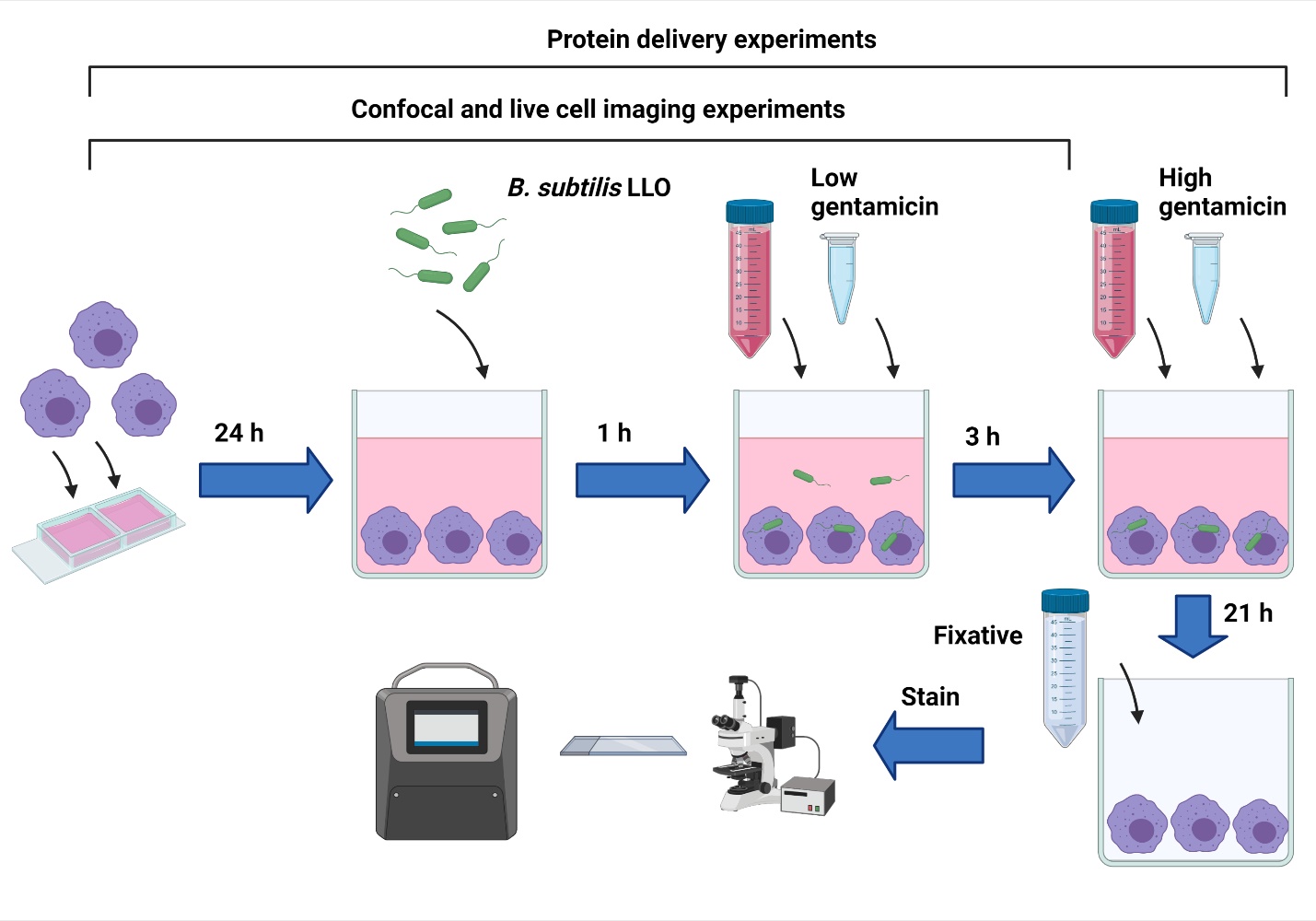


Supplementary Fig. 1: Diagram of method to deliver engineered bacteria and analyze interaction with host cells

General approach for co-*incubating* *B. subtilis* LLO with host J774A.1 cells and timeline for the interaction. EES are allowed to incubate with J774A.1 cells for 1 h with the appropriate inducer depending on condition before a low concentration of gentamicin is added to eliminate extracellular EES. Incubation continues for 3 more hours with a second inducer before a high concentration of gentamicin is used to eliminate intracellular EES. Incubation is continued for 21 h to determine impact on host cells by imaging or other methods such as flow cytometry and cytokine/chemokine profiling.

**
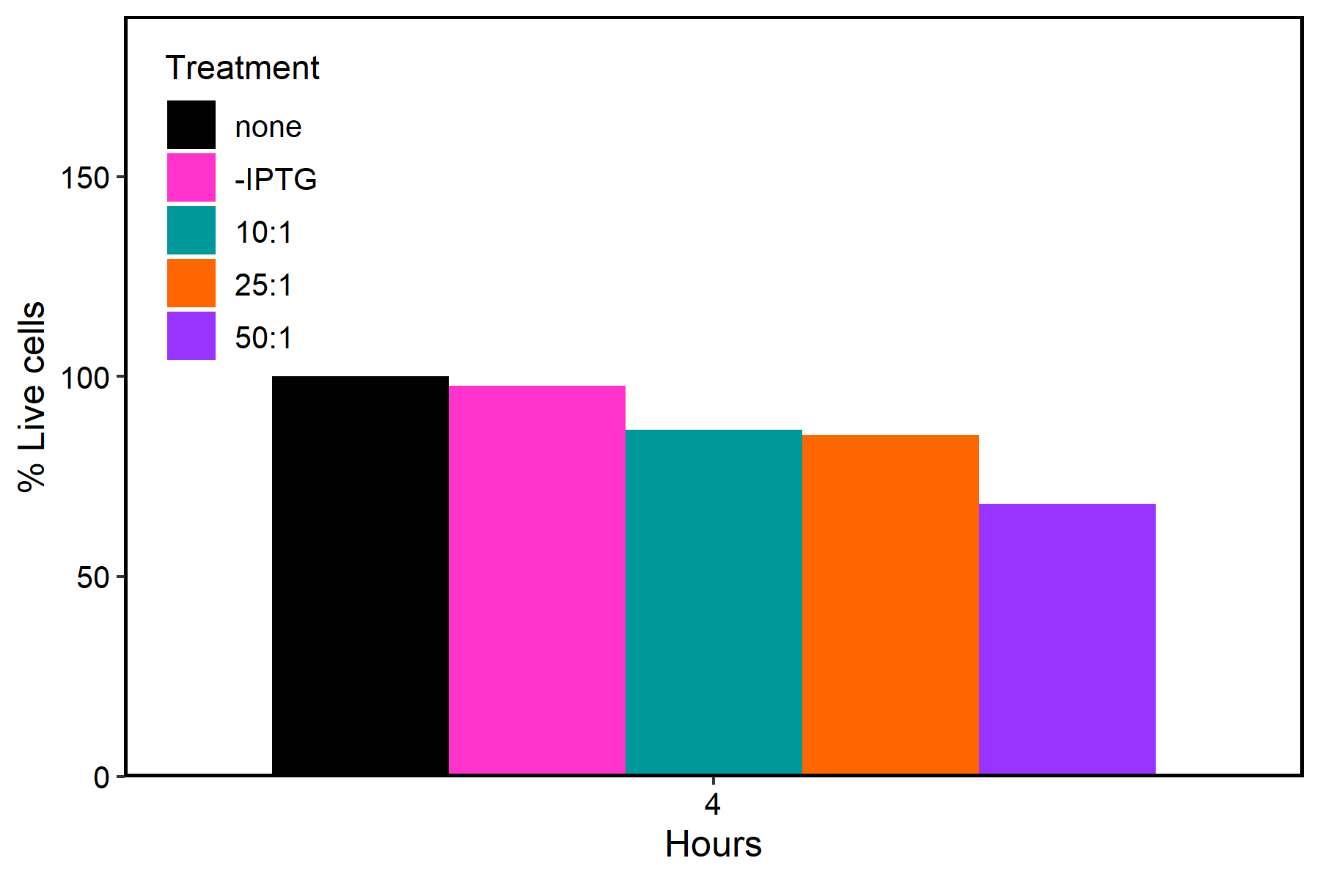
**

Supplementary Fig. 2: Flow cytometry analysis of viable J774A.1 cells

J774A.1 cells were treated with the LLO strain without IPTG (25:1 MOI) and with IPTG at different MOIs for 4 h J774A.1 were analyzed for viability using flow cytometry. Experiment was performed with one biological replicate (n = 1) to test for the same trend as the MTS assay. Number of events>20,000 cells.

Supplementary Table 1: Quantification table of LLO strain interaction with J774A.1 cells

Quantification of LLO strain presence within J774A.1 cells using a 25:1 MOI without IPTG, and different MOI with IPTG at 1 and 2 h post LLO-strain addition.


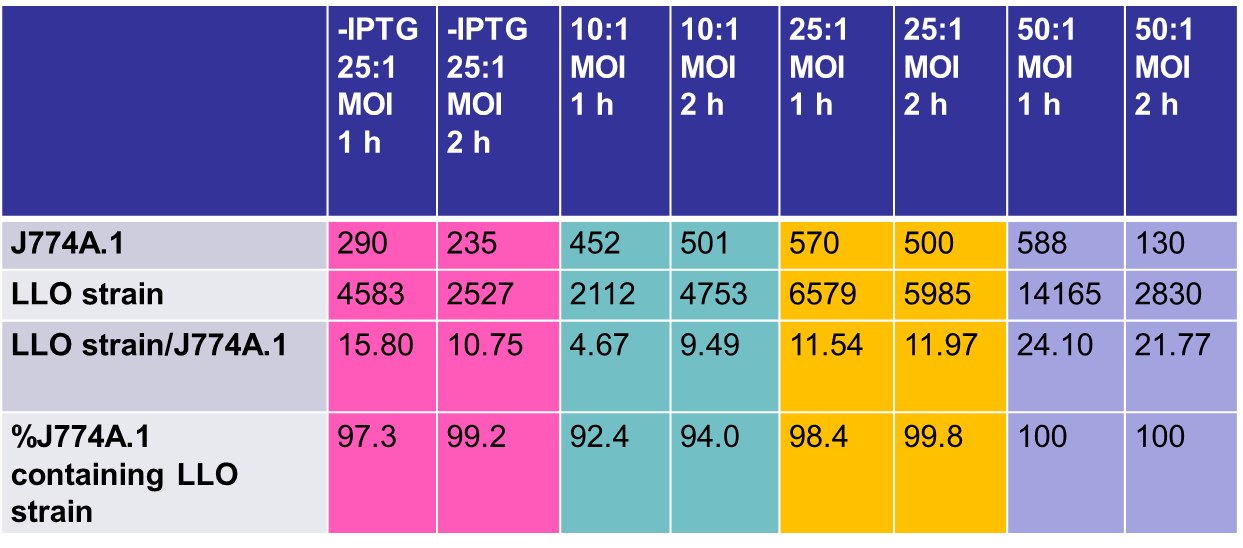


**
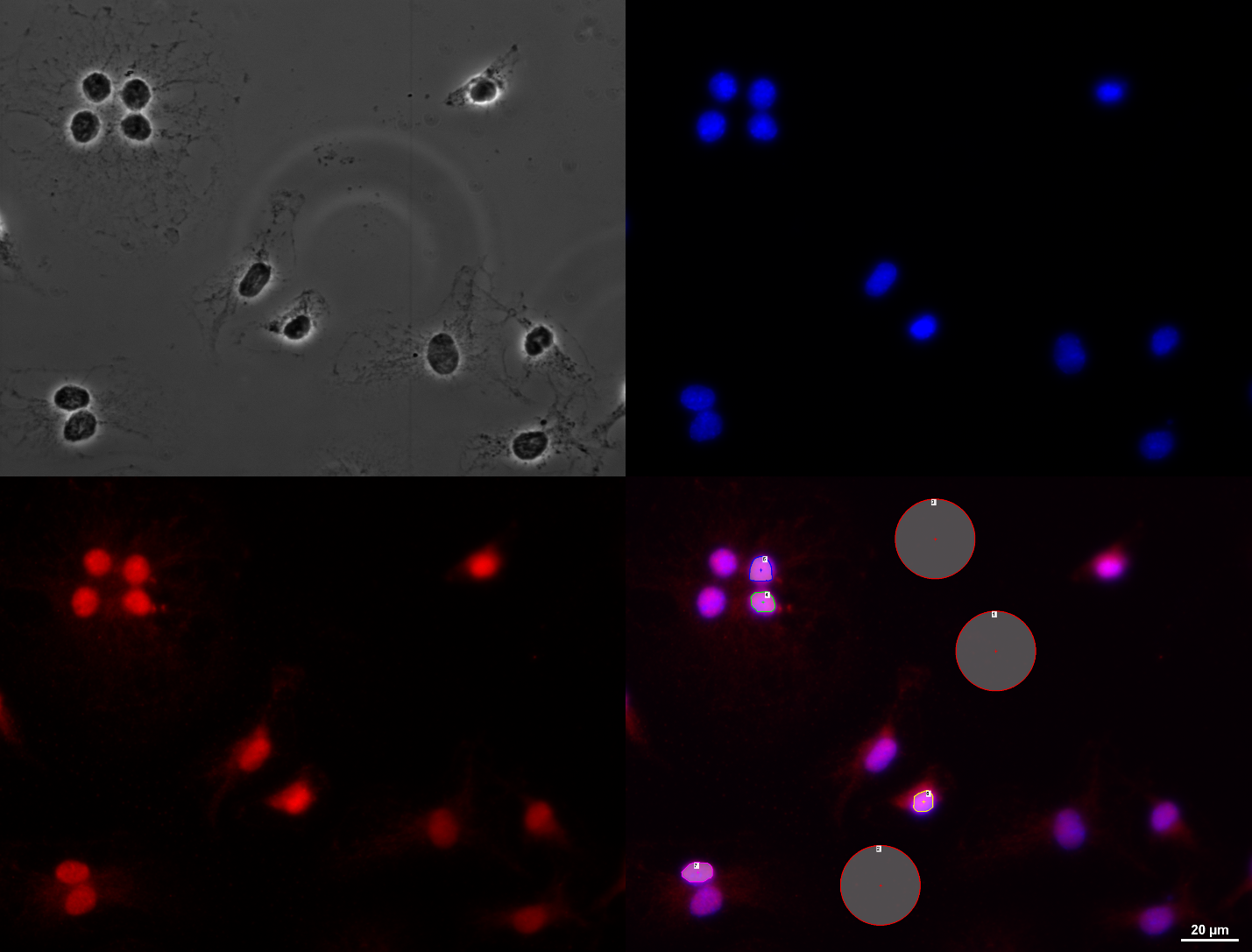
**

Supplementary Fig. 3: Quantification method for calculating SNR

Representative images showing quantification of β-gal fluorescence in nucleus by SNR. Phase contrast (upper left), Hoechst 33342 nuclear stain (upper right), Cy5 secondary antibody for β-gal (lower left) and overlay of Hoechst 3342 and Cy5 (lower right) as panels. Larger circles denoting background signal (noise) and smaller signals drawn around nuclei for quantification to generate SNR (lower right).


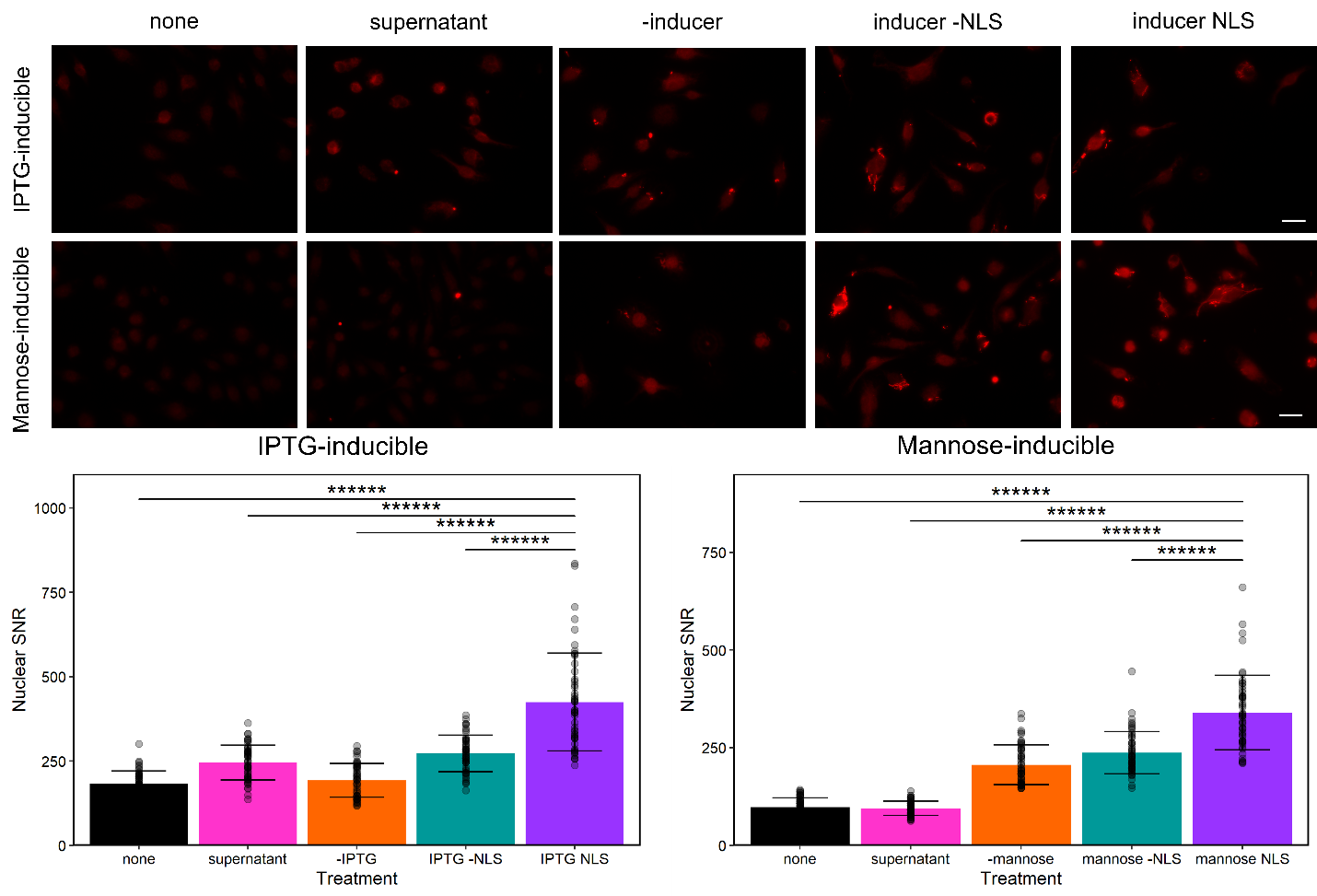


Supplementary Fig. 4. Intracellular LLO-*lacZ* secretes β-gal to nuclei of J774A.1 cell using IPTG- and mannose-inducible systems

Fluorescence of nuclei in J774A.1 cells with no LLO strain (none), J774A.1 cells incubated with β-gal collected as supernatant from induced LLO-*lacZ*-NLS (supernatant), J774A.1 cells incubated with uninduced LLO-*lacZ*-NLS (-mannose), J774A.1 cells incubated with induced LLO-*lacZ*-no NLS (mannose -NLS) and J774A.1 cells incubated with induced LLO-*lacZ*-NLS (mannose NLS). Plotted data is mean ± SD from n = 50 random individuals in a representative experiment; ******p<0.000001. Scale bars = 20 µm.

**
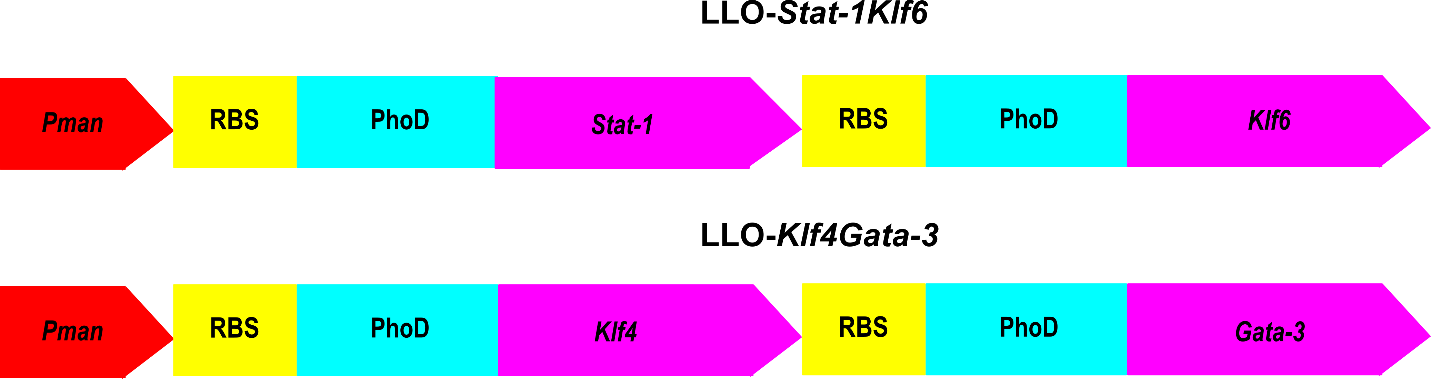
**

Supplementary Fig. 5: Visualization of polarization operons created for EES expression

The operons for polarization were designed to be transcriptionally controlled by the mannose promoter (*Pman*). Then a Gram-positive ribosomal binding site (RBS) and secretion peptide (PhoD) were synthesized in front of each gene in both operons.

**
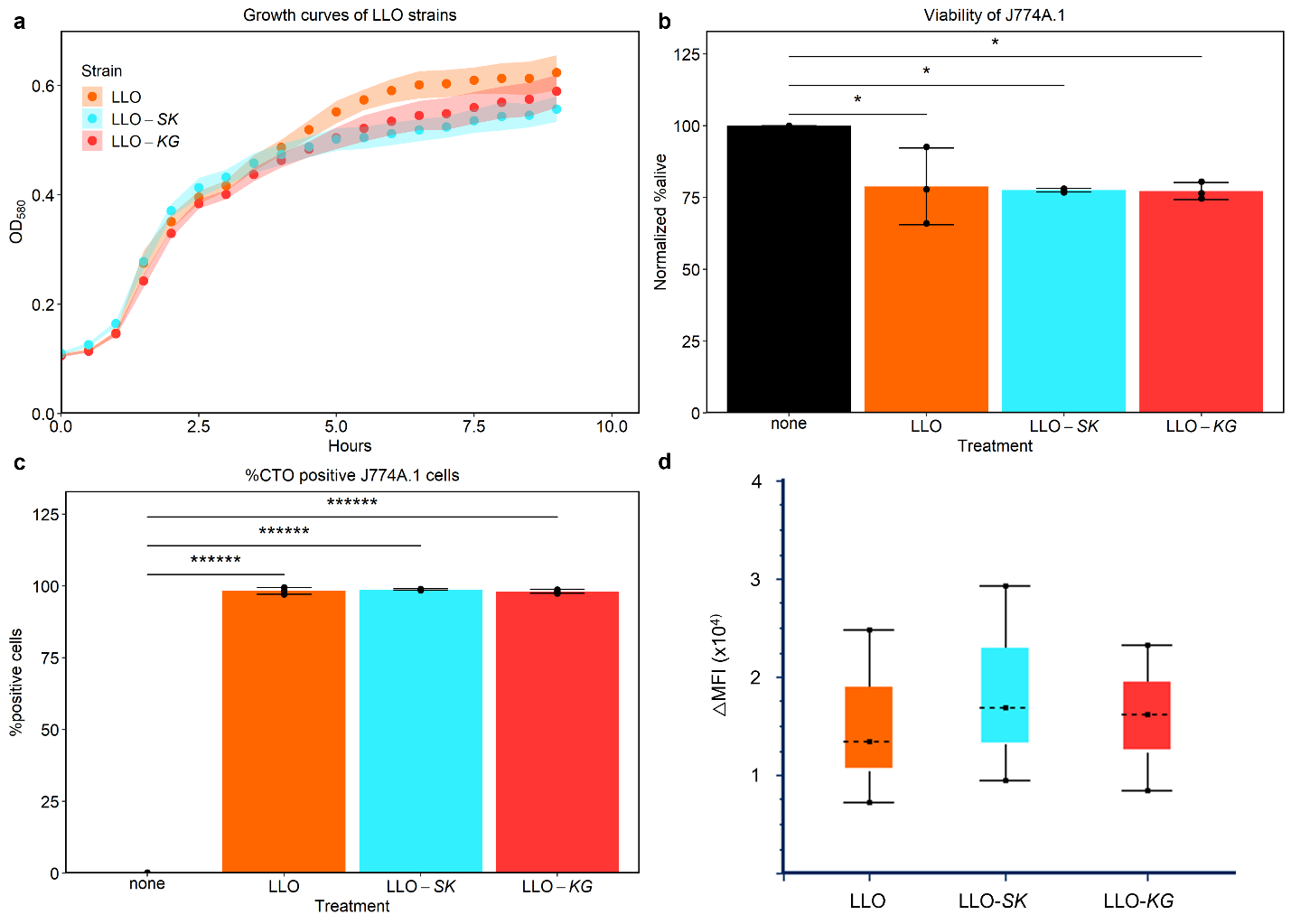
**

Supplementary Fig. 6: Characterization of engineered *B. subtilis* LLO strains activity in growth and interaction with J774A.1 cells

Growth rates of the LLO strain (LLO), LLO-*SK* and LLO- *KG* were evaluated for any differences (a). Flow cytometry was used to identify the strains impact on J774A.1 viability (b, percent viable cells) and ability to escape phagosomal destruction and persist in J774A.1 cells (c, percent cells that are positive for fluorescent LLO strains). Box and whisker plots (d) demonstrate distribution of CTO (CellTracker Orange) positive J774A.1 cells, relating to the presence of fluorescently labeled strains within the cells. ΔMFI represents the change in CTO intensity in the CTO positive population. Data is mean ± SD from either n = 3 biological replicate or n = 3 biological with n = 8 technical for growth curve; *p<0.05, ******p<0.000001.

**
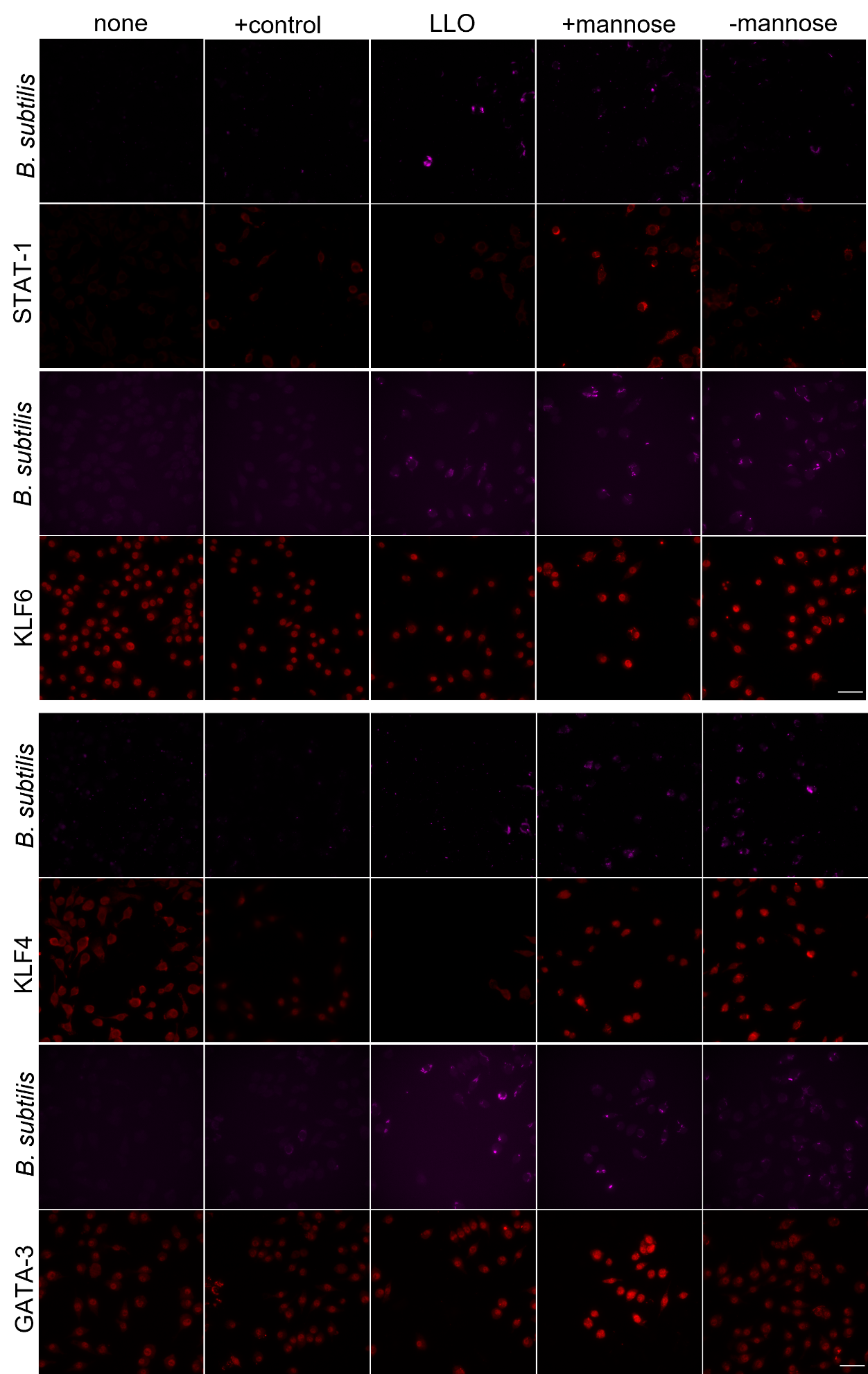
**

**
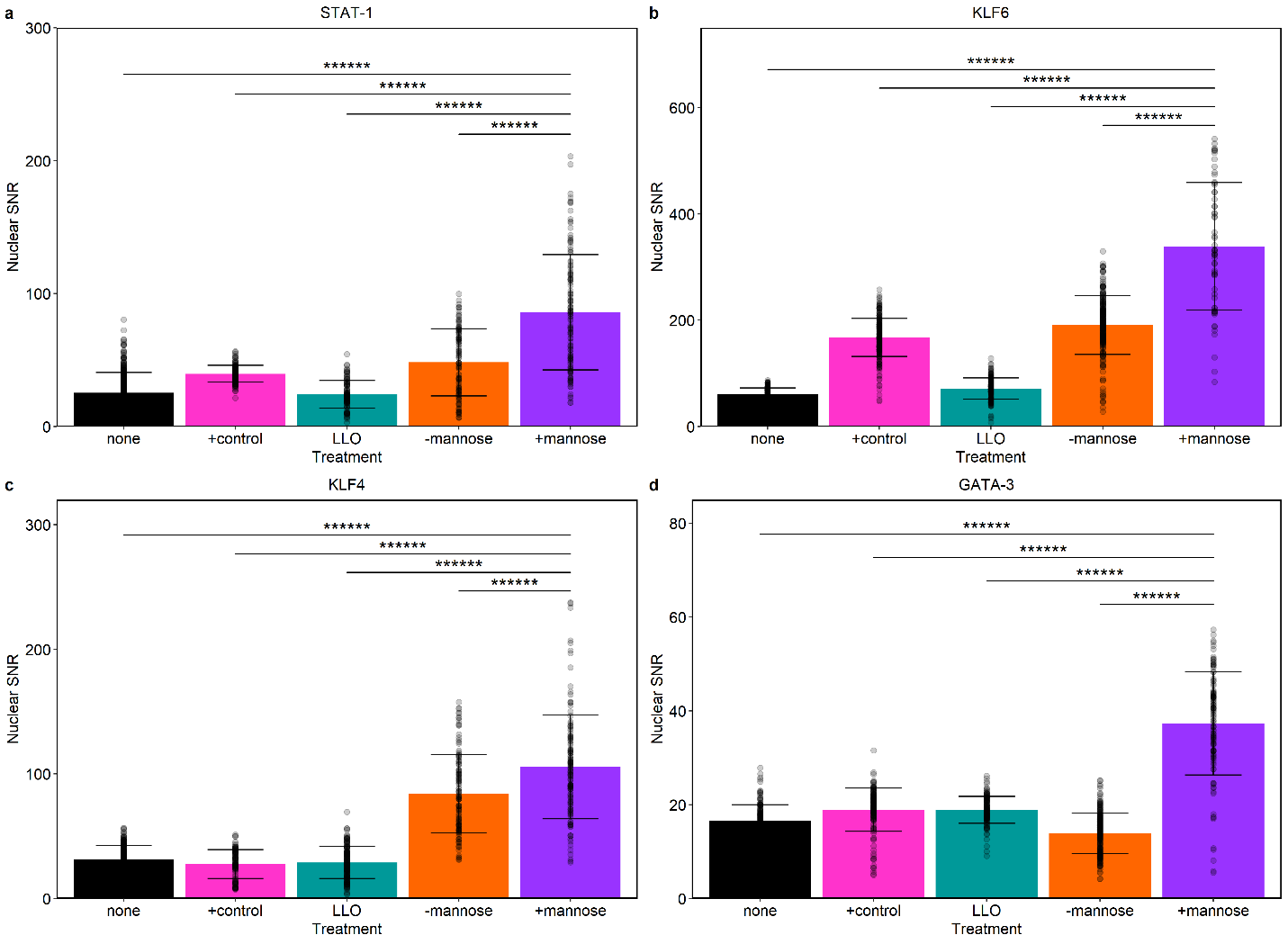
**

Supplementary Fig. 7. Engineered *B. subtilis* LLO production and secretion of mammalian transcription factors

Fluorescence microscopy (top) identifies *B. subtilis* (magenta) and transcription factors (red) in J774A.1 cells treated with nothing (none), positive control (STAT-1/KLF6: LPS and IFN-γ or KLF4/GATA-3: IL-4 and IL-13), LLO strain (LLO), LLO-*SK* or LLO-*KG* not induced (-mannose) or induced (+mannose). Each row dynamic range was scaled to the image with the highest fluorescence intensity to compare conditions. Scale bars = 50 µm with all images taken at magnification = 400X. Quantification of nuclear fluorescence (bottom) quantified from each transcription factor: STAT-1 (a), KLF6 (b), KLF4 (c) and GATA-3 (d). Plotted data is mean ± SD from n = 158.4 +/- 63.6 (SD) random individuals in a representative experiment; ******p<0.000001.

**
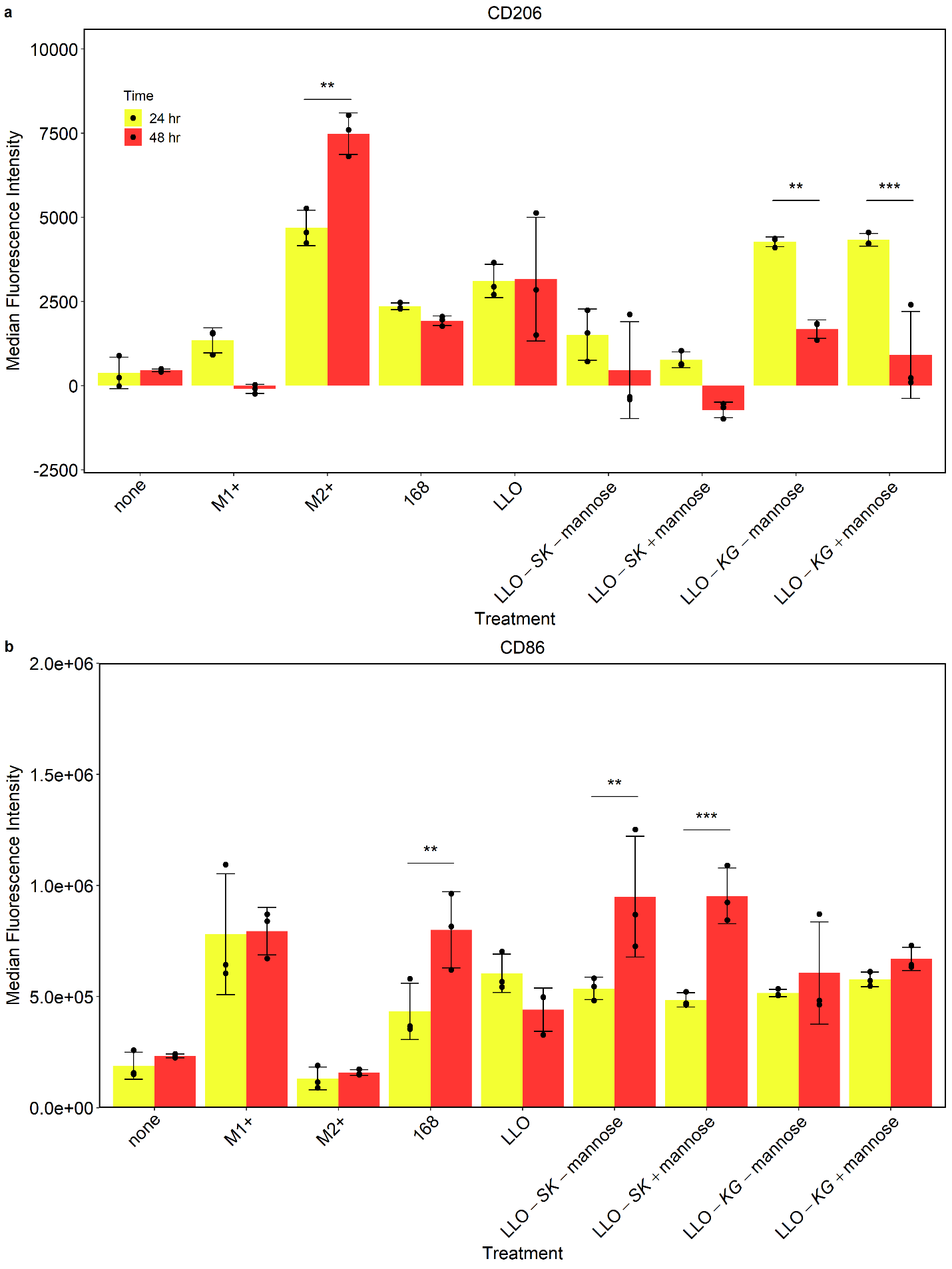
**

**Supplementary Fig. 8. Flow cytometry reveals differences in impact of engineered *B. subtilis* LLO strains on J774A.1 cell marker expression between 24 and 48 h time points**

Median fluorescence intensity of CD206 (a) and CD86 (b), comparing cell marker expression between 24 and 48 h time points. J774A.1 cells were treated with nothing (none), LPS and IFN-γ (M1+), IL-4 and IL-13 (M2+), LLO strain (LLO), LLO-*SK* with and without mannose (LLO-*SK* -mannose, LLO-*SK­* +mannose) and LLO-*KG* with and without mannose (LLO-*KG* -mannose, LLO-*KG* +mannose. Plotted data is mean ± SD from n = 3 biological replicates in representative experiment; **p<0.01, ***p<0.001.

)

. Data is mean ± SD; **p<0.01, ***p<0.001.


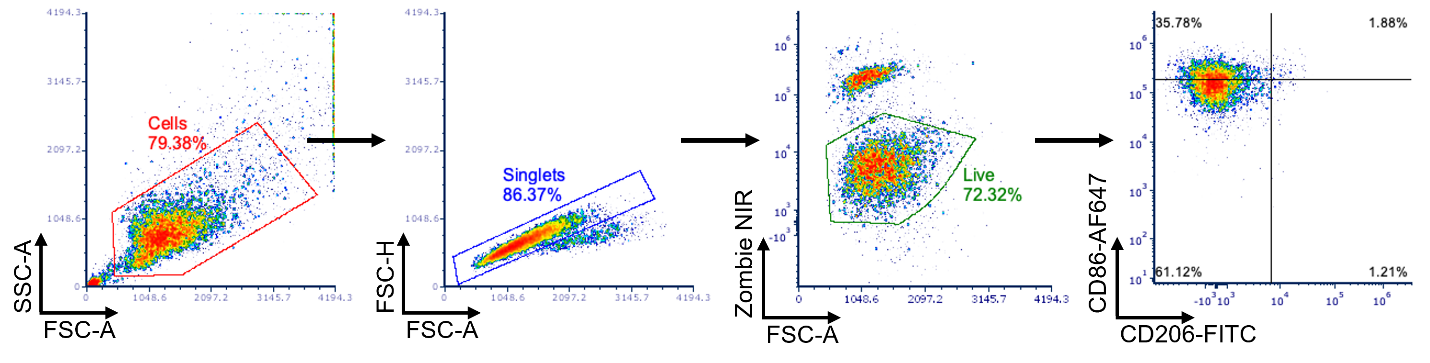


**Supplementary Fig. 9. Gating strategy for flow cytometry**

Flow density dot plots were used to identify the live population of cells followed by calculation of percent positive cells and median fluorescence intensity. FSC-A and SSC-A were used to identify the cell population which was then gated for singlets using FSC-A and FSC-H. Live cells were gated from the singlet population as Zombie NIR negative. CD86-AF647 and CD206-FITC gates were set using single stained controls.


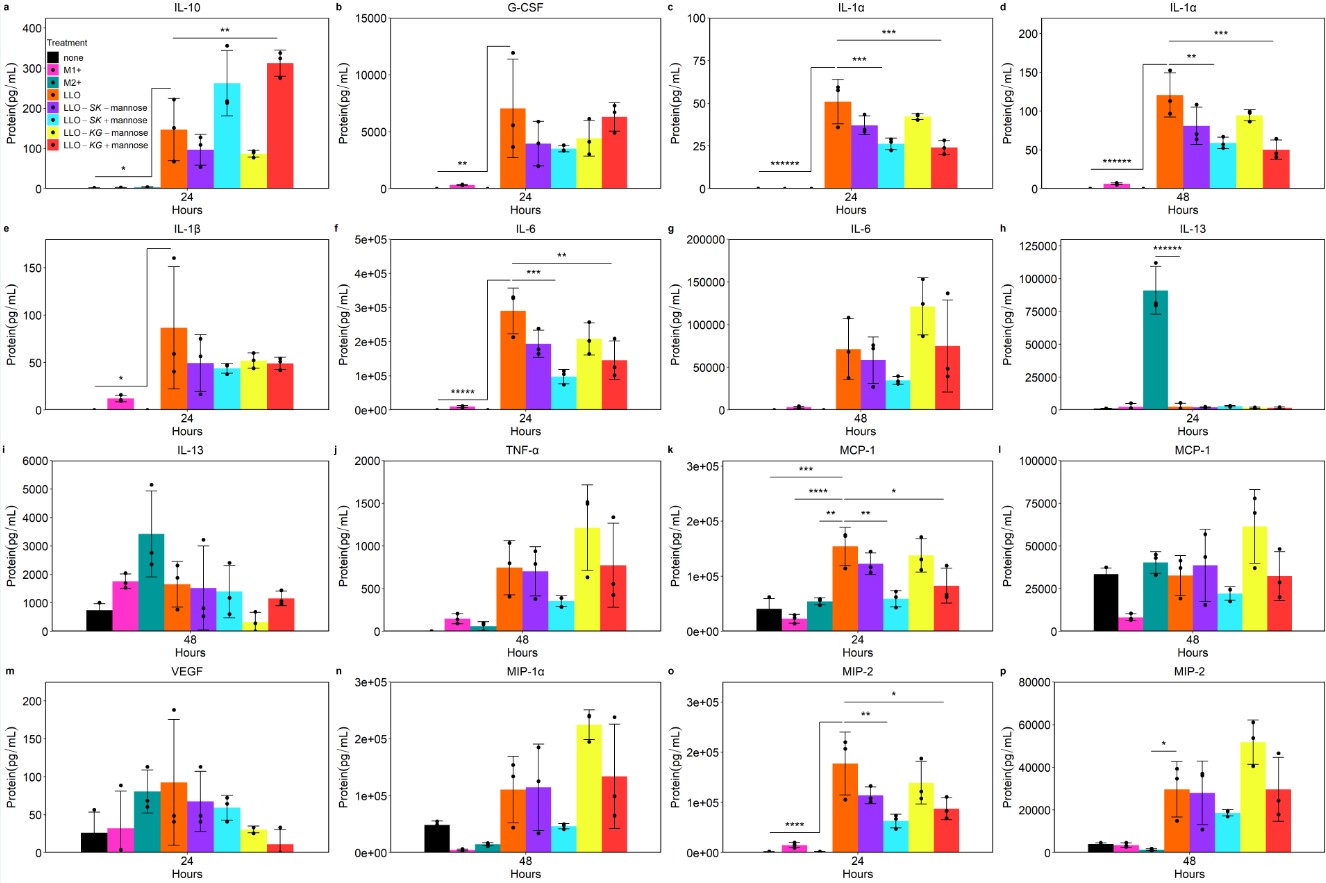


**Supplementary Fig. 10. Remainder of Luminex cytokine profiling assay characterizing EES impact on J774A.1 cell cytokine and chemokine expression**

Cytokine protein concentration was quantified after J774A.1 cells were treated with nothing (none), LPS and IFN-γ (M1+), IL-4 and IL-13 (M2+), LLO strain (LLO), LLO-*SK* with and without mannose (LLO-*SK* -mannose, LLO-*SK­* +mannose) and LLO-*KG* with and without mannose (LLO-*KG* -mannose, LLO-*KG* +mannose) at 24 and 48 h post initial treatment. Data is mean ± SD from n = 3 biological replicates; *p<0.05, **p<0.01, ***p<0.001, ****p<0.0001, *****p<0.00001, ******p<0.000001. Significance shown is comparing LLO strain condition to all other conditions.
