# Supplementary material for: Engineered endosymbionts that alter mammalian cell surface marker, cytokine and chemokine expression
Source: Commun Biol. 2022 Aug 30;5:888. doi: 10.1038/s42003-022-03851-6 (PMC9427783; doi:10.1038/s42003-022-03851-6)
Supplement: Supplementary file 3 — Description of Additional Supplementary Files [file 42003_2022_3851_MOESM3_ESM.docx]

Supplementary Data 1: List of oligos that were used to build DNA constructs in study

The primers used for cloning in this study are mentioned first with forward (F) primer and reverse (R) primer pairs mentioned together. Gblocks used for inserting secretion peptide with and without nuclear localization signal (NLS) for β-gal secretion are mentioned next. Finally, synthesized transcription factor genes as Gblocks or as a custom gene are mentioned last.
