# Supplementary material for: Engineered endosymbionts that alter mammalian cell surface marker, cytokine and chemokine expression
Source: Commun Biol. 2022 Aug 30;5:888. doi: 10.1038/s42003-022-03851-6 (PMC9427783; doi:10.1038/s42003-022-03851-6)
Supplement: Supplementary file 7 — Reporting summary [file 42003_2022_3851_MOESM7_ESM.pdf]

nature portfolio

Corresponding author(s): Cody S. Madsen, Ashley V. Makela, Emily M Greeson, Jonathan W. Hardy, Christopher H. Contag

Last updated by author(s): Apr 5, 2022

Reporting Summary

Nature Portfolio wishes to improve the reproducibility of the work that we publish. This form provides structure for consistency and transparency in reporting. For further information on Nature Portfolio policies, see our Editorial Policies and the Editorial Policy Checklist.

Statistics

For all statistical analyses, confirm that the following items are present in the figure legend, table legend, main text, or Methods section.

n/a	Confirmed	
☐	☒	The exact sample size (*n*) for each experimental group/condition, given as a discrete number and unit of measurement
☐	☒	A statement on whether measurements were taken from distinct samples or whether the same sample was measured repeatedly
☐	☒	The statistical test(s) used AND whether they are one- or two-sided<br>*Only common tests should be described solely by name; describe more complex techniques in the Methods section.*
☒	☐	A description of all covariates tested
☐	☒	A description of any assumptions or corrections, such as tests of normality and adjustment for multiple comparisons
☐	☒	A full description of the statistical parameters including central tendency (e.g. means) or other basic estimates (e.g. regression coefficient) AND variation (e.g. standard deviation) or associated estimates of uncertainty (e.g. confidence intervals)
☐	☒	For null hypothesis testing, the test statistic (e.g. *F*, *t*, *r*) with confidence intervals, effect sizes, degrees of freedom and *P* value noted<br>*Give P values as exact values whenever suitable.*
☒	☐	For Bayesian analysis, information on the choice of priors and Markov chain Monte Carlo settings
☒	☐	For hierarchical and complex designs, identification of the appropriate level for tests and full reporting of outcomes
☒	☐	Estimates of effect sizes (e.g. Cohen's *d*, Pearson's *r*), indicating how they were calculated

Our web collection on statistics for biologists contains articles on many of the points above.

Software and code

Policy information about availability of computer code

Data collection	NIS-Elements AR Software (Nikon), LAS-X software, Fiji (ImageJ), NIS elements BR 5.21.02 software, Luminex 200 analyzer instrument, FCSExpress, R (x64 4.0.4), Prism software 9.2.0 (GraphPad), Cytek Aurora flow cytometer
Data analysis	Statistical analyses were performed using Prism software (9.2.0, GraphPad Inc., La Jolla, CA). Statistical tests are identified for each method. Data are expressed as mean +/- standard deviation; p<.05 was considered a significant finding. Plotting was performed using R version 4.0.4 with the following packages: ggplot2, dplyr, reshape2, ggsignif and ggpubr.

For manuscripts utilizing custom algorithms or software that are central to the research but not yet described in published literature, software must be made available to editors and reviewers. We strongly encourage code deposition in a community repository (e.g. GitHub). See the Nature Portfolio guidelines for submitting code & software for further information.

Data

Policy information about availability of data

All manuscripts must include a data availability statement. This statement should provide the following information, where applicable:

- Accession codes, unique identifiers, or web links for publicly available datasets
- A description of any restrictions on data availability
- For clinical datasets or third party data, please ensure that the statement adheres to our policy

All raw data, EES constructs and R scripts will be made available upon request by the corresponding author. Plasmids used to produce EES constructs will be submitted to Addgene after manuscript publication. All R scripts were written with established packages.

Field-specific reporting

Please select the one below that is the best fit for your research. If you are not sure, read the appropriate sections before making your selection.

☒ Life sciences ☐ Behavioural & social sciences ☐ Ecological, evolutionary & environmental sciences

For a reference copy of the document with all sections, see nature.com/documents/nr-reporting-summary-flat.pdf

Life sciences study design

All studies must disclose on these points even when the disclosure is negative.

Sample size	Sample sizes were guided by literature that had looked at treatments on the same cell line; literature for methods being performed such as chemokine/cytokine profiling, flow cytometry, MTS assay, fluorescence quantification; and by appropriate replicates for statistical tests being performed.
Data exclusions	No data was excluded.
Replication	Biological replication was used in accordance with appropriate statistical tests chosen. All replications were included in final data analysis and worked with reproducibility. Live cell imaging was performed multiple times but only random areas within one experiment was used due to software failure due to amount of data generated during the experiment in other replications.
Randomization	Randomization was used in all imaging studies. Random areas around a well in a treatment were selected to perform analyses. For studies involving measuring an output of the entire population, randomization was not used such as cytokine/chemokine analysis and flow cytometry.
Blinding	Blinding is not necessary due to not collecting subjective data regarding a treatment.

Reporting for specific materials, systems and methods

We require information from authors about some types of materials, experimental systems and methods used in many studies. Here, indicate whether each material, system or method listed is relevant to your study. If you are not sure if a list item applies to your research, read the appropriate section before selecting a response.

Materials & experimental systems

n/a	Involved in the study	
☐	☒	Antibodies
☐	☒	Eukaryotic cell lines
☒	☐	Palaeontology and archaeology
☒	☐	Animals and other organisms
☒	☐	Human research participants
☒	☐	Clinical data
☒	☐	Dual use research of concern

Methods

n/a	Involved in the study	
☒	☐	ChIP-seq
☐	☒	Flow cytometry
☒	☐	MRI-based neuroimaging

Antibodies

Antibodies used	Rabbit anti-Subtilisin antibody (Antibodies-online , ABIN459110)<br>Goat anti-Rabbit IgG (H+L) Secondary Antibody [DyLight 650] (Novus Biologicals, Cat# NBP1-76058, RRID:AB_11029095)<br>Anti-LAMP1 antibody [1D4B] (AbCam, Cat# ab25245, RRID:AB_449893)<br>Goat anti-Rat IgG (H+L) Cross-Adsorbed Secondary Antibody, Alexa Fluor 555 (ThermoFisher, Cat# A-21434, RRID:AB_2535855)<br>anti-β-galactosidase (E. coli) antibody-rabbit (BioRad, AHP1292GA)<br>TruStain FcX™ PLUS (anti-mouse CD16/32) Antibody (Biolegend, Cat# 156603, RRID:AB_2783137)<br>Alexa Fluor® 647 anti-mouse CD86 Antibody (Biolegend, Cat# 105020, RRID:AB_493464)<br>Brilliant Violet 421™ anti-mouse CD206 (MMR) Antibody (Biolegend, Cat# 141717, RRID:AB_2562232)<br>anti-STAT1 antibody :: Rabbit STAT1 Antibody (MyBioSource, MBS125754)<br>anti-KLF6 antibody :: Rabbit anti-Human, Mouse KLF6 Polyclonal Antibody (MyBioSource, MBS8307089)<br>anti-KLF4 antibody :: Rabbit Kruppel Like Factor 4, Gut (KLF4) Polyclonal Antibody (MyBioSource, MBS2014661)<br>anti-GATA3 antibody :: Rabbit GATA3 Polyclonal Antibody (MyBioSource, MBS8204267)
Validation	All antibodies were chosen specifically because of the manufacturer confirming functionality using multiple approaches and research articles being cited that demonstrated usage and functionality of the antibodies under conditions reported by the manufacturer.

Eukaryotic cell lines

Policy information about cell lines

Cell line source(s)	J774A.1 monocyte/macropahge cell line
Authentication	ATCC purchase and immediate usage
Mycoplasma contamination	Cells were tested for mycoplasma throughout use and remained negative.
Commonly misidentified lines (See ICLAC register)	*Name any commonly misidentified cell lines used in the study and provide a rationale for their use.*

Flow Cytometry

Plots

Confirm that:

☒ The axis labels state the marker and fluorochrome used (e.g. CD4-FITC).

☒ The axis scales are clearly visible. Include numbers along axes only for bottom left plot of group (a 'group' is an analysis of identical markers).

☒ All plots are contour plots with outliers or pseudocolor plots.

☒ A numerical value for number of cells or percentage (with statistics) is provided.

Methodology

Sample preparation	Samples were prepared for staining by resuspending 1x106 cells in 100 µl 1X PBS in a 96-well round bottom plate. Samples were first incubated with Zombie NIR viability dye (1:750, Biolegend, San Diego, CA, USA; Cat# 423105) for 15min at RT in the dark. Cells were washed once with flow buffer, followed by incubation with TruStain FcX™ PLUS (anti-mouse CD16/32) Antibody (Biolegend, Cat#156603; 1.25 µl/sample) for 10min on ice. Alexa Fluor® 647 anti-mouse CD86 Antibody (2.5 ul/sample; Biolegend; Cat#105020) was then added and incubated for 20 min at RT in the dark. Cells were washed twice with flow staining buffer and fixed with 4% paraformaldehyde for 10min in the dark. Cells were permeabilized (0.3% TritonX-100 in flow wash buffer) followed by incubated with Brilliant Violet 421™ anti-mouse CD206 (MMR) Antibody (1.25 µl/sample; Biolegend; Cat# 141717) for 20 min at RT in the dark. Cells were washed twice with flow buffer and resuspended in a final volume of 100 µl for flow cytometry analysis using the Cytek Aurora spectral flow cytometer (Cytek Biosciences, CA, USA).
Instrument	Cytek Aurora
Software	Flow cytometry data was analyzed with the software FCSExpress (DeNovo Software, CA, USA).
Cell population abundance	A single cell line was cultured.
Gating strategy	FSC-A and SSC-A were used to identify the cell population (cells). FSC-A and FSC-H gated on cells was used for doublet discrimination (singlets). Zombie NIR viability dye was used to identify the live cell population (negative population). The median fluorescence intensity histograms were gated on live cells. Percent positive cells were gated using the appropriate FMO controls.

☒ Tick this box to confirm that a figure exemplifying the gating strategy is provided in the Supplementary Information.
